# Alterations in gut immunological barrier in SARS-CoV-2 infection and their prognostic potential

**DOI:** 10.3389/fimmu.2023.1129190

**Published:** 2023-03-15

**Authors:** Gerasimos Eleftheriotis, Efthymios P. Tsounis, Ioanna Aggeletopoulou, Periklis Dousdampanis, Christos Triantos, Athanasia Mouzaki, Markos Marangos, Stelios F. Assimakopoulos

**Affiliations:** ^1^ Division of Infectious Diseases, Department of Internal Medicine, Medical School, University of Patras, Patras, Greece; ^2^ Division of Gastroenterology, Department of Internal Medicine, University Hospital of Patras, Patras, Greece; ^3^ Laboratory of Immunohematology, Division of Hematology, Department of Internal Medicine, Medical School, University of Patras, Patras, Greece; ^4^ Department of Renal Diseases, “Agios Andreas” Patras State General Hospital, Patras, Greece

**Keywords:** COVID-19, gut barrier, cytokines, interferon, defensins, microbiome, cytokine storm

## Abstract

Although coronavirus disease 2019 (COVID-19) is primarily associated with mild respiratory symptoms, a subset of patients may develop more complicated disease with systemic complications and multiple organ injury. The gastrointestinal tract may be directly infected by SARS-CoV-2 or secondarily affected by viremia and the release of inflammatory mediators that cause viral entry from the respiratory epithelium. Impaired intestinal barrier function in SARS-CoV-2 infection is a key factor leading to excessive microbial and endotoxin translocation, which triggers a strong systemic immune response and leads to the development of viral sepsis syndrome with severe sequelae. Multiple components of the gut immune system are affected, resulting in a diminished or dysfunctional gut immunological barrier. Antiviral peptides, inflammatory mediators, immune cell chemotaxis, and secretory immunoglobulins are important parameters that are negatively affected in SARS-CoV-2 infection. Mucosal CD4+ and CD8+ T cells, Th17 cells, neutrophils, dendritic cells, and macrophages are activated, and the number of regulatory T cells decreases, promoting an overactivated immune response with increased expression of type I and III interferons and other proinflammatory cytokines. The changes in the immunologic barrier could be promoted in part by a dysbiotic gut microbiota, through commensal-derived signals and metabolites. On the other hand, the proinflammatory intestinal environment could further compromise the integrity of the intestinal epithelium by promoting enterocyte apoptosis and disruption of tight junctions. This review summarizes the changes in the gut immunological barrier during SARS-CoV-2 infection and their prognostic potential.

## Introduction

Although the primary target of severe acute respiratory syndrome coronavirus-2 (SARS−CoV−2) is type II alveolar epithelial cells, the virus can also infect gastrointestinal mucosal cells by binding to angiotensin-converting enzyme 2 (ACE2) and its cofactor, transmembrane serine protease 2 (TMPRSS2), both of which are widely expressed on the surface of enterocytes. Notably, ACE2 expression is higher in intestinal cells of the ileum and colon than in lung cells (1). In experimental models, intranasal inoculation of SARS-CoV-2 resulted in disruption of gut barrier integrity as a consequence of systemic release of proinflammatory mediators (2). Therefore, the virus can cause either direct damage to ACE-2-expressing intestinal epithelial cells or indirect damage through a systemic hyperinflammatory response (2, 3). Recombination is a common mechanism in coronaviruses that allows them to resist selective pressure and adapt to new habitats. Recombination events with other gut-targeting coronaviruses could potentially enhance SARS-CoV-2 virulence and tropism for the gastrointestinal tract (4). Indeed, up to 24% of COVID-19 patients develop gastrointestinal symptoms, including diarrhea, abdominal discomfort, nausea, vomiting, and loss of appetite (3, 5). Notably, COVID-19 patients may even develop severe duodenitis and present with gastrointestinal bleeding requiring red blood cell transfusion (6). Immunohistochemical staining of these biopsies was positive for SARS-CoV-2 spike protein, suggesting that duodenitis developed as a result of direct enterocyte invasion by SARS-CoV-2; *in situ* hybridization also provided evidence of active viral replication (6). SARS-CoV-2 infection is associated with multifactorial impairment of the gut barrier, as it has deleterious effects on all of its critical aspects of defense, which consist of a balance between the gut microbiota (biological barrier), intestinal epithelial cells and their junctions (mechanical barrier), and gut-associated immune cells, immunoglobulins, and cytokine production (immune barrier). Previous studies have shown that the integrity of the intestinal barrier is significantly impaired in COVID-19 patients, as evidenced by various surrogate markers. Giron et al. (7) demonstrated that severe COVID-19 is associated with higher plasma levels of zonulin, indicating profound disruption of tight junction homeostasis, as well as increased levels of lipopolysaccharide (LPS)-binding protein (LBP) and β-glucan, which are reliable markers of bacterial and fungal translocation, respectively. Importantly, serum markers of tight junction permeability and microbial translocation were significantly associated with circulating proinflammatory mediators such as IL-6, suggesting that systemic inflammation is triggered to some extent by gut barrier disruption. In addition, intestinal fatty acid binding protein (I-FABP), a protein synthesized by mature enterocytes responsible for fatty acid turnover and used as a biomarker of intestinal injury, was measured in the urine of 283 patients hospitalized for COVID-19 (8, 9). Urinary I-FABP levels were significantly elevated compared with controls and remained high in patients’ samples two weeks after hospitalization; levels were even higher in patients with critical illness than in milder cases (8). Another study found that serum levels of zonula occludins-1 (ZO-1), a marker of structural and functional integrity of the paracellular barrier, were significantly elevated in patients with COVID-19 pneumonia but were not predictive of progression to severe respiratory failure (10). In addition, numerous studies have shown significant changes in the composition of the gut microbiota in patients with COVID-19. A recent meta-analysis detailed the changes in gut microbiota composition during SARS-CoV-2 infection (11). In several studies, changes in the gut microbiome were also closely associated with the clinical severity of COVID-19, suggesting a prognostic role in such patients (12–24). The present review focuses specifically on COVID-19 associated changes in the gut immunological barrier and their prognostic potential.

## SARS-CoV-2-mediated changes in the intestinal immunological barrier

### Intestinal inflammation and fecal calprotectin

Fecal calprotectin has also been studied in detail in COVID-19 patients because it is produced predominantly by neutrophils that migrate to and are activated in the intestine, making it a reliable marker of bowel inflammation in other conditions such as inflammatory bowel disease (25). Ojetti et al. (26) showed that high fecal calprotectin levels in SARS-CoV-2 infected individuals are an independent risk factor for the development of COVID-19 pneumonia, while data from other studies indicate that fecal calprotectin levels are positively correlated with serum IL-6, degree of hypoxemia, and days of hospitalization (27–29). Although there is no correlation between gastrointestinal symptoms and fecal calprotectin, an increase in this parameter has a better predictive value for progression of severe disease compared with C-reactive protein (30). This finding possibly suggests that the increase in fecal calprotectin associated with SARS-CoV-2 infection is due in part to chemotaxis of immune cells into to the gastrointestinal tract and hypoxic intestinal damage rather than intestinal inflammation and destruction of enterocytes (29, 30). In addition, the role of serum calprotectin was also investigated and was found to be an effective marker for predicting the future status of SARS-CoV-2-infected individuals (31). The strong correlation of serum calprotectin with poor clinical outcomes highlights the potential value of this marker in identifying COVID-19 patients at high risk for disease progression (31).

### Changes in immune cells

Data are also available on changes in immune cells in the gastrointestinal mucosa of patients infected with SARS-CoV-2. Mass cytometric analysis of intestinal tissue from deceased individuals with COVID-19 revealed leukocytic infiltration consisting of monocytes, CD11b+ macrophages, CD11c+ dendritic cells (DCs), natural killer (NK) cells, and B cells (32). Similar results were obtained from lung tissue, suggesting that these organs are the epicenter of the immune response during SARS-CoV-2 infection (32). Moreover, analysis of duodenal biopsies after the onset of COVID-19 symptoms in patients with macroscopically normal mucosa who underwent endoscopy for other reasons (e.g., upper abdominal pain) revealed increased numbers of CD68+, CD14+ macrophages, CD11c+ DCs, mucosal CD4+ T cells, and intraepithelial CD8+ T cells (33). The aforementioned intraepithelial lymphocytes were antigen-experienced and exhibited a CD8+ effector cell phenotype (CD45RA+, CD27-) (33). An increase in intraepithelial lymphocytes in intestinal biopsies was still observed one month after SARS-CoV-2 infection (34). Exhaustion/depletion of CD4+ T cells, a hallmark of HIV infection, is also observed in SARS-CoV-2 infection. The resulting dysregulation of CD4+ T cells in the gut may contribute to intestinal epithelial barrier dysfunction and leaky gut, which promotes systemic inflammation (35). Accordingly, IL-17 producing Th17 cells are overactivated in SARS-CoV-2 infection (36).

#### Imbalance of cytokines and inflammatory mediators

##### Dysregulation of interferon responses

Invasion of SARS-CoV-2 into intestinal cells leads to increased expression of type I and III IFN and other proinflammatory cytokines, such as IL-8 and IL-12 (37, 38). All types of IFN can activate the JAK/STAT pathway. Type I IFN can be secreted by many cell types, especially plasmacytoid DCs. SARS-CoV-2 has developed several strategies to evade immune surveillance by attenuating type I and III IFN responses (39). This effect is mediated by a SARS-CoV-2 membrane protein that inhibits the formation of a multiprotein complex responsible for the phosphorylation and subsequent activation of IFN regulatory factor (IRF) 3 (39). IRF3 activation is a prerequisite for IFN transcription and synthesis. The SARS-CoV-2 accessory protein ORF9b is another molecule that blocks the IRF3 activation pathway (40). ORF9b also inhibits IFN gene expression by interacting with the stimulator of IFN genes (STING); STING can recruit TANK binding kinase 1 (TBK1), one of the IRF3 phosphorylators (40). In addition, ORF9b targets the translocase of outer mitochondrial membrane 70 (TOMM70), which is located on the mitochondrial membrane and functions as a receptor for mitochondrial antiviral signaling protein (MAVS). MAVS is also involved in the IRF3 phosphorylation pathway. Therefore, ORF9b downregulates type I IFN production by interfering with the interaction between TOMM70 and MAVS (41, 42). However, overexpression of TOMM70 can overcome ORF9b-mediated inhibition and restore IFN-β expression (41).

After pretreatment of human intestinal cell lines with IFN-β and human colon organoids with IFN-β1 and type III IFN, respectively, a protective effect against SARS-CoV-2 infection was observed, resulting in a significantly milder infection (43, 44). However, the antiviral activity of type III IFN against SARS-CoV-2 in the gut is stronger and more durable (45). Of note, infection of colon organoids with SARS-CoV-2 resulted in upregulation of type III IFN but not type I IFN, despite the ability of these organoids to produce both types in response to viral infection (44, 46). Conversely, depletion of the type III IFN receptor resulted in increased SARS-CoV-2 infectivity, viral genome replication, and virion production (44, 45).

Although neutralizing autoantibodies to type I IFN have been previously demonstrated in humans and are a universal finding, particularly in autoimmune polyendocrine syndrome type 1 (APS-1), they have not been associated with increased prevalence or severity in viral infections (47–50). SARS-CoV-2 infection appears to be an exception; in an international cohort study, 19 of 22 APS-1 patients with COVID-19 were hospitalized, and 11 of 22 required mechanical ventilation (51). Pre-existing neutralizing IFN type I antibodies in the serum of previously healthy individuals represented a major risk factor for severe COVID-19 (51). Moreover, their prevalence increases with age; during SARS-CoV-2 infection, 10.2% of patients with life-threatening disease, almost exclusively men, had pre-existing neutralizing IFN-I autoantibodies and low or undetectable serum IFN-α levels, whereas no such antibodies were detected in patients with mild infection (52, 53). In addition, COVID-19 patients with type I IFN antibodies had significantly higher viral loads than patients without these antibodies (54). Similar results were obtained in other studies investigating the presence of neutralizing IFN-I antibodies in patients with COVID-19 in the intensive care unit (55, 56). Such autoantibodies were detected in 9.5% to 18% of these patients; 87% to 92.3% of them were men (55, 57). In contrast, non-neutralizing IFN-I antibodies are common in critically ill non-COVID-19 patients and do not affect clinical outcome (58). The aforementioned data provide an additional explanation for the increased likelihood of severe disease in elderly men; notably, this group of individuals accounts for approximately 20% of COVID-19 deaths (53).

Furthermore, impairment of IFN-I-dependent immunity caused by any mechanism can lead to severe COVID-19 symptoms. Loss-of-function variants of genes such as IRF3, IRF7, IFN-α receptor, and Toll-like receptor 3 (TLR3) were detected in 3.5% of individuals with life-threatening COVID-19 and no history of other severe infections, whereas no patient with mild or asymptomatic disease carried these variants; all of these variants resulted in disproportionately low IFN I production in response to SARS-CoV-2 (59).

#### Changes in other inflammatory mediators

Following intranasal infection with SARS-CoV-2, cytokines such as IL-4, IL-1β, TNF-α, IL-17A, and other inflammatory mediators are initially produced in gastrointestinal tissues (2). In parallel, upregulation of the anti-inflammatory IL-10 and inhibition of the pro-inflammatory IL-1β and IFN-γ can be induced by inoculation in the digestive tract (2). A gut-on-a-chip model of SARS-CoV-2 infection provided further evidence for the release of cytokines in the digestive tract (60). In particular, the IL-6 and TNF genes and C-X-C motif chemokine ligand 10 (CXCL10), a chemoattractant for NK cells and T cells and a monocyte inducer, were significantly upregulated (60). Gene set enrichment analysis in pluripotent stem cells derived from small intestinal epithelial cells or intestinal organoids from SARS-CoV-2 infection models also revealed increased expression of IL-1β, IL-6, CXCL10, C-C motif chemokine ligand 5 (CCL5, chemoattractant for monocytes), and significant upregulation of IL-6 and the nuclear factor-κB (NF-κB) pathway (61, 62). Quantification of cytokines in the stool of patients hospitalized for COVID-19 revealed higher IL-8, IL-18, and lower IL-10 levels (63, 64).

In contrast, treatment of human colon tissue samples with short-chain fatty acids (SCFAs), metabolites capable of reducing pro-inflammatory mediators such as IL-6, IL-12, and IFN-γ, showed no effect on cell permeability; however, treatment with SCFAs showed a modest, albeit significant, effect in reducing the expression of the type III IFN receptor, interferon lambda receptor 1 (IFNLR1), and the serine protease TMPRSS2 (56, 65). TMPRSS2 is a membrane-bound protein that has been shown to promote SARS-CoV-2 infection in enteroids by supporting virus-enterocyte fusion (66). Of note, depletion of the gut microbiota after antibiotic administration did not affect mortality in a mouse model of SARS-CoV-2 infection although colonic concentrations of IL-17 and CXCL2 were significantly increased (67). In contrast, administration of remdesivir to SARS-CoV-2-infected intestinal epithelial cells resulted in reduced induction of the IL-1β, IL-6, CXCL10, and CCL5 genes (62).

Another protein inversely correlated with serum IL-6 levels during SARS-CoV-2 infection is soluble mucosal addressin cell adhesion molecule (sMAdCAM), which is expressed by gut endothelial venules to induce migration of immune cells into the intestine (68, 69). At the same time, sMAdCAM levels were lower in COVID-19 patients compared to healthy controls or convalescent subjects, suggesting that normalization of sMAdCAM levels may signify the restoration of mucosal homeostasis and highlighting its role as an important systemic and gut homing parameter that needs to be monitored for better therapeutic guidance and prophylactic intervention in COVID-19 (68, 69).

#### Disruption of antimicrobial peptide production

Regarding the antiviral response of the gastrointestinal tract, Paneth cells and neutrophils are also capable of producing the immunomodulatory proteins, defensins. Defensins are important members of the antimicrobial peptide (AMP) family with diverse immunoregulatory functions and a broad spectrum of antimicrobial and antiviral effects. These proteins act as chemoattractants and activators for immature DCs, monocytes, and naive T cells (70, 71). During SARS-CoV-2 infection, α-defensin 5, a lectin-like protein that can recognize lipids and glycoproteins, shields the ACE2 receptor and prevents SARS-CoV-2 binding (72). Although SARS-CoV-2 has a higher affinity for the ACE2 receptor than α-defensin 5 (72), intestinal α-defensin 5 has a protective effect because it is highly abundant in the digestive tract. Consequently, α-defensin 5 levels are elevated before infection (72). Indeed, administration of α-defensin 5 to a cell line model after infection resulted in no antiviral response, whereas pretreatment with α-defensin 5 showed a beneficial effect (73). Of note, β-defensin 1 production is increased in later stages of SARS-CoV-2 infection due to intestinal hypoxia mediated by hypoxia-inducible factor 1α (74).

#### Dysregulation of secretory IgA production

The immunologic barrier of the gut is also strengthened by secretory immunoglobulin A (sIgA) produced by mucosal lymphoid tissues, including gut-associated lymphoid tissue (GALT). Dimeric sIgAs are the predominant mucosal antibodies and form an essential component of the immunologic barrier (75). Commensal microorganisms play a central role in controlling IgA class switching and effective antibody production. Indeed, the number of functional IgA-secreting B cells is drastically reduced in germ-free animal models (76, 77). The predominance of sIgA in the intestine is likely another explanation for the attenuated gut inflammation compared with lung tissue; IgA dimers are able to inactivate toxins or pathogens without inducing inflammation because they cannot bind and activate complement (78). An *in vitro* study examining the neutralizing ability of IgG and IgA from B cells of COVID-19 convalescent subjects found that dimeric IgA was much more effective than IgA monomers or IgG in neutralizing SARS-CoV-2 (79).

## SARS-CoV-2-mediated gut microbiome and immunological changes

The constant interaction of immune cells with the gut microbiome maintains the balance between tolerance to beneficial bacteria and eradication of pathogenic species (80). A complex, dynamic, and bilateral interaction between the gut microbiome and COVID-19 has been described (81). The gut microbiome of patients with SARS-CoV-2 infection exhibits significant alterations, possibly due to a severe systemic inflammatory response. The mechanisms underlying COVID-19-related dysbiosis are still unclear. However, interactions between the ACE2 receptor and SARS-CoV-2 have been associated with alterations in the composition of the gut microbiota by impairing the secretion AMPs. The function of the amino acid transporter B0AT1, which mediates intestinal uptake of tryptophan, is dependent on the ACE2 pathway (82). Tryptophan modulates the production of AMPs *via* the mammalian target of rapamycin (mTOR) pathway (83). Therefore, the deficiency of tryptophan caused by ACE-2 blockade may decrease the production of AMPs and disrupt the intraluminal microbial species. Commensal bacteria also play a critical role in mucosal homeostasis by modulating the expression of ACE2 in the gut (84). Secretion of proinflammatory cytokines, particularly TNF-α, during respiratory tract infections has a dynamic anorexigenic effect *via* hypothalamic activity. The decrease in fiber and caloric intake disrupts the composition of the gut microbiota and the synthesis of its metabolites, which in turn strongly influence the transcriptional “training” of innate immune cells (85).

A recent meta-analysis detailed the changes in the gut microbiota during SARS-CoV-2 infection (11). At the phylum level, dysbiosis is characterized by a reduction in the ratio of *Firmicutes* to *Bacteroidetes*. In particular, COVID-19 is associated with fewer butyrate-producing bacterial species, including *Faecalibacterium* and *Roseburia* (11, 15, 86). The genus *Roseburia* is closely associated with colon motility and mucosal tissue integrity and has a crucial anti-inflammatory effect by regulating IL-10 synthesis (87). Several other beneficial genera, including *Eubacterium*, *Alistipes*, and *Bifidobacterium*, are also reduced in COVID-19 patients (11). *Bifidobacterium* strains mediate robust antimicrobial and antiviral activity, which is balanced by promotion of Treg-mediated responses and induction of tolerogenic DC phenotypes (88).

Alterations in the gut microbiome have also been closely associated with clinical severity of COVID-19 in several studies, suggesting a prognostic role in such patients (12–24). Bacterial genera with significant prognostic value included *Eubacterium, Ruminococcus, Faecalibacterium, Bacteroides, Lactobacillus, Clostridium, Roseburia*, and *Bifidobacterium* (12–24). An increase in the dominant genus *Enterococcus* and a decrease in the families *Ruminococcaceae* and *Lachnospiraceae* have been reported in severe COVID-19 cases admitted to the intensive care unit (21).

**Figure 1:**
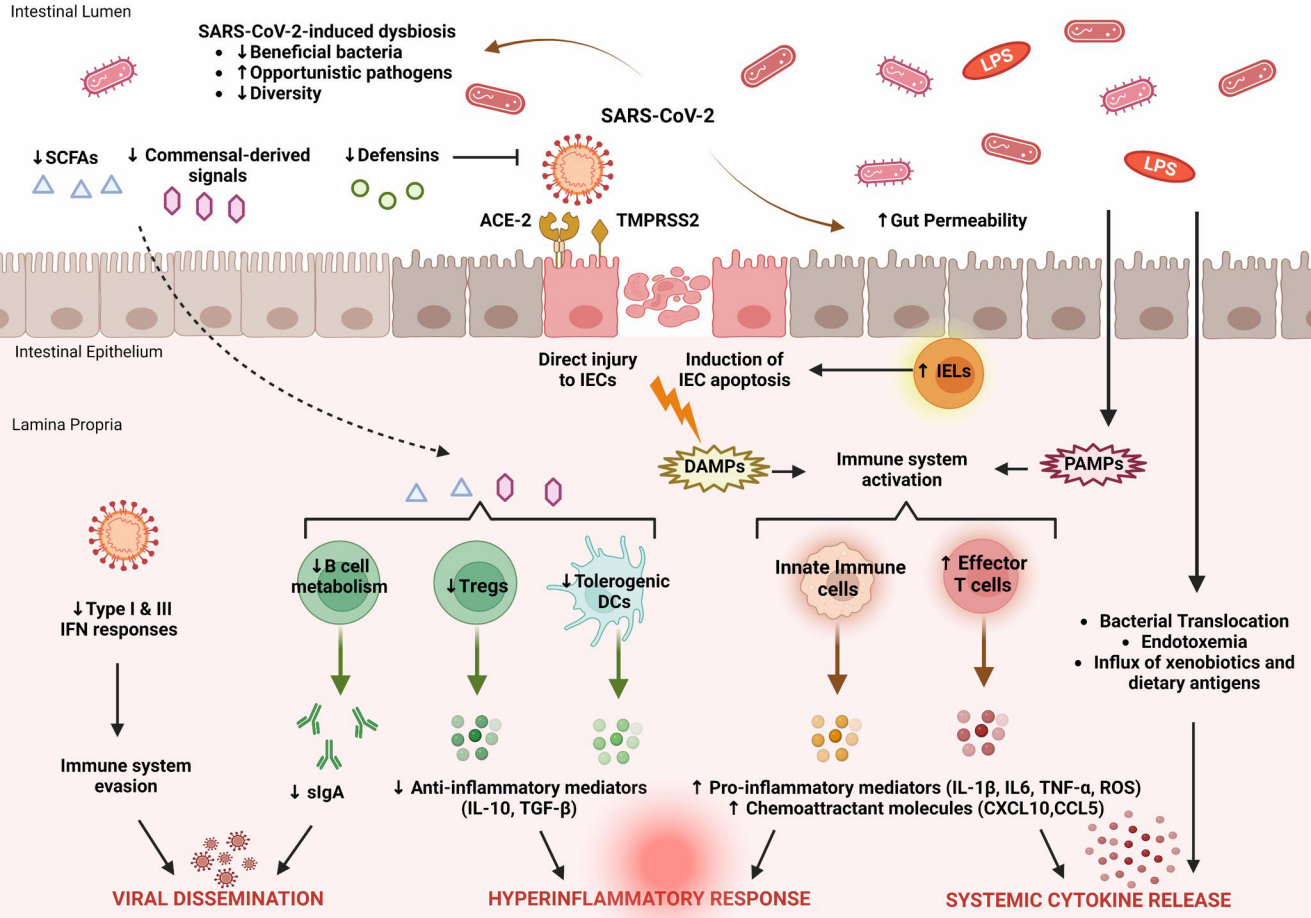
Key features of intestinal immune barrier disruption during SARS-CoV-2 infection. SARS-CoV-2 infection is associated with profound alterations in the intestinal microflora, manifested by decreased species diversity, depletion of symbiotic microorganisms, and prevalence of pathogenic species. Signals and metabolites derived from the intestinal flora, such as short-chain fatty acids (SCFAs), play an important role in controlling mucosal immunity by promoting T regulatory cell (Treg) responses and the activity of tolerogenic dendritic cells (DCs). This immunoregulatory environment, rich in anti-inflammatory mediators (IL-10, TGF-β), is significantly impaired by SARS-CoV-2. As a result, B cell metabolism and maturation are severely impaired, leading to exhaustion of effective plasma cells that produce secretory dimeric immunoglobulin A (sIgA), which is essential for viral containment. The proliferation of SARS-CoV-2 is also facilitated by its ability to evade recognition by the immune system by interfering with type I and type III IFN signaling. SARS-CoV-2 exerts either direct cytopathic effects on intestinal epithelial cells (IECs) expressing ACE2 and TMPRSS2 receptors or indirect immune-mediated injury. During COVID-19, the expression of several antimicrobial peptides, including defensins, is dysregulated, which increases the infectivity of SARS-CoV-2. In addition, recruitment of intraepithelial lymphocytes (IELs) accelerates IEC apoptosis. The release of damage-associated molecular patterns (DAMPs) due to cell injury and the influx of pathogen-associated molecular patterns (PAMPs) as a result of increased gut permeability lead to immune activation. Macrophages/monocytes, neutrophils, and other cells of the innate immune system secrete large amounts of proinflammatory mediators (IL-1β, IL-6, TNF-α, ROS) and chemokines (CCL5, CXCL10) that cause recruitment of additional immune cells and prime effector T cells. In parallel, disruption of the intestinal barrier facilitates bacterial translocation, endotoxemia, and dissemination of other gut-derived stimuli that contribute to systemic hyperinflammatory responses and cytokine release syndrome, leading to severe COVID-19.

The changes in the gut microbiota in patients with COVID-19 should be considered as a dynamic process (81). Emerging evidence suggests that the regulatory functions of the gut microbiota effectively support recovery from SARS-CoV-2 infection. The main features of intestinal immune barrier disruption during SARS-CoV-2 infection are shown in **Figure 1**.

## The prognostic potential of gut immunologic barrier alterations in SARS-CoV-2 infection

SARS-CoV-2 affects multiple systems, including the gastrointestinal tract and gut barrier integrity. COVID-19 is associated with multifaceted disruption of the various components of the mucosal immune barrier, and the extent of these changes reflects the severity of the underlying disease. In particular, SARS-CoV-2 is able to evade the innate immune response by disrupting interferon signaling. The expression pattern of several cytokines in the mucosal compartment is severely affected, essentially leading to the recruitment and activation of additional immune cells that support this proinflammatory milieu. In addition, the production and release of antimicrobial peptides and secretory IgA, which are important regulators of intestinal immune integrity, are impaired. As a result, profound alterations of the gut microbiome and metabolome occur, characterized by depletion of symbiotic species and dominance of pathogenic microorganisms. The main features of SARS-CoV-2-induced dysregulation of the intestinal immune barrier are shown in **Figure 1**.

The prognostic value of various parameters related to the gut immunological barrier was evaluated. A strong association was described between certain prognostic factors and disease severity, poor prognosis, hospitalization, or mortality due to SARS-CoV-2. **Table 1** summarizes important parameters related to the gut immunologic barrier that have been studied as prognostic markers for severity and progression of SARS-CoV-2 infection. It is unlikely that a single index of gut immunologic barrier function can independently predict progression of COVID-19. Alternatively, the development and validation of a prognostic scoring system that incorporates the most robust immunologic parameters and combines them with additional epidemiologic, clinical, and laboratory data may provide the best results. Further prospective studies with larger numbers of participants are warranted to identify markers of gut barrier dysfunction that could help identify high-risk COVID-19 patients who require early or enhanced support.

**Table 1:** Key gut immunologic barrier parameters that serve as prognostic markers for severity of SARS-CoV-2 infection and poor prognosis.

Parameter studied	Endpoints of the study	Results of the study	Refs.
Serum calprotectin	Identification of the association between serum calprotectin, neutrophil secretory proteins, and other inflammatory mediators with COVID-19 severity and outcome.	Correlation between serum calprotectin levels and disease severity. Significant increase in serum calprotectin along with worsening of clinical symptoms of the disease.	(31)
Fecal calprotectin	Identification of an association between fecal calprotectin and the severity of pulmonary manifestations caused by COVID-19.	Significant association between COVID-19 pneumonia and high levels of fecal calprotectin. Higher calprotectin levels in women compared with men, suggesting that men with high calprotectin have a worse prognosis.	(26)
sMAdCAM	Cross-sectional and longitudinal study of sMAdCAM at different stages of disease progression after SARS-CoV-2 infection.	sMAdCAM is considered a possible integrated marker of inflammation and homeostatic immune migration. Association of sMAdCAM with COVID-19 disease progression and generation of potentially neutralizing antibody responses against SARS-CoV-2.	(68)
Autoantibodies to type I IFNs	Evaluation of immunological and clinical characteristics of APS-1 patients during the course of SARS-CoV-2 infection.	Pre-existing neutralizing autoantibodies to type I IFNs pose an increased risk of life-threatening COVID-19 pneumonia at any age.	(51)
	High-throughput autoantibody screening for autoantibodies against 2,770 extracellular and secreted proteins in SARS-CoV-2- infected individuals.	Pathologic role of exoproteome-targeted autoantibodies in SARS-CoV-2 infection and differential impact on immune function and clinical course.	(54)
	Evaluation of the prevalence of IFN I autoantibodies and their association with clinical disease progression.	In the presence of IFN-I autoantibodies, there is an increased risk of developing severe COVID-19.	(57)
Type I IFN variants	Assessment of the role of monogenic inborn errors in the development of life-threatening COVID-19.	Inborn errors of IRF7- and TLR3-dependent type I IFN immunity cause life-threatening COVID-19 pneumonia in patients without prior severe infection.	(59)
Cytokines in stool samples	Evaluation of cytokines, inflammatory markers, viral RNA, microbiome composition, and antibody responses in stool samples from hospitalized COVID-19 patients.	Increased fecal levels of IL-8 and lower fecal levels of IL-10 in COVID-19 hospitalized patients. Fecal IL-23 is higher in more severe COVID-19. Intestinal virus-specific IgA responses are associated with more severe disease.	(63)
Secretory IgA antibodies	Characterization of IgA response to SARS-CoV-2 after COVID-19 diagnosis.	Responses against dimeric IgA may be a valuable tool for protection against SARS-CoV-2 and for vaccine efficacy.	(79)
Gut microbiota	Association of intestinal microflora alterations with COVID-19 and its severity.	Poor prognosis is associated with: ↑*Bacteroides*, ↑*Parabacteroides*, ↑*Clostridium,* ↑*Bifidobacterium*, ↑*Ruminococcus*, ↑*Campylobacter*, ↑*Rothia*, ↑*Enterococcus,* and ↑*Aspergillus* spp. ↓*Roseburia*, ↓*Eubacterium,* ↓*Lachnospira*, ↓*Faecalibacterium*, and ↓*Firmicutes/Bacteroidetes* ratio.	(11)

I-FABP, intestinal fatty-acid binding protein; sMAdCAM, soluble mucosal addressin cell adhesion molecule; IFN, interferon; APS-1, autoimmune polyendocrine syndrome type 1; IRF7, IFN regulatory factor 7, toll-like receptor 3, TLR3; IgA, immunoglobulin A. Upward arrows are used to indicate an increase, and downward arrows indicate a decrease.

## Concluding remarks

SARS-CoV-2 infection is associated with significant disruption of intestinal immunological homeostasis and impairs mucosal immune cell function and production of signaling molecules. SARS-CoV-2-induced gut dysbiosis could drive many of these immunological changes through commensal-derived signals and metabolites that maintain a continuous dialog between the mucosal immune system and the gut microflora. Conversely, dysregulation of intestinal immune cells and overproduction of proinflammatory cytokines could compromise the integrity of intestinal epithelial cells (apoptosis induction) and their connections (disruption of tight junctions), further promoting gut barrier dysfunction. These alterations contribute to the breakdown of intestinal barrier integrity, which may subsequently lead to translocation of microbes and endotoxins from the intestinal lumen into the systemic circulation, promoting a hyperinflammatory response associated with distant organ dysfunction and the development of a “viral sepsis syndrome” (89). The importance of gut immunologic barrier alterations in COVID-19 is underscored by several studies demonstrating their prognostic potential. Features of intestinal immune barrier failure occur early in the course of infection and correlate well with the severity of COVID-19, suggesting that immune barrier dysfunction is not only a bystander but an active participant in fueling exuberant immune responses and systemic inflammation. Further clinical studies are needed to explore the role of appropriate biomarker-based immunologic therapies in improving gut barrier function, which could lead to an expansion of therapeutic options against COVID-19.

## Author contributions

SFA, conceptualization; GE, PD, and CT, review of bibliography; GE, ET, and IA, preparation of draft; ET and IA, preparation of figure; GE, PD, CT, MM, SFA, and AM, preparation of revised version of manuscript, review and editing of manuscript; all authors were responsible for revising the manuscript for important intellectual content. All authors contributed to the article and approved the submitted version.
